# The invisible enemy: A systematic review and meta-analysis of maternal smokeless tobacco use as a risk factor for low birth weight

**DOI:** 10.1371/journal.pone.0312297

**Published:** 2024-12-30

**Authors:** Akanksha Mahajan, Bhawna Gupta, Michael Tong

**Affiliations:** 1 Faculty of Medicine, Nursing and Health Sciences, Monash University, Melbourne, Victoria, Australia; 2 Department of Public Health, Torrens University, Melbourne, Victoria, Australia; 3 National Centre for Epidemiology and Population Health, Australian National University, Canberra, Australia; Mahidol University, THAILAND

## Abstract

**Introduction:**

Smokeless tobacco use is a growing public health concern, with potential adverse implications for foetal outcomes if consumed during pregnancy. Birth weight is an important predictive measure for health outcomes of a child throughout their lifespan. Despite extensive literature, it is unclear whether smokeless tobacco consumption during pregnancy has an adverse effect on birth weight. Hence, this review was conducted to determine whether an association exists between maternal smokeless tobacco consumption during pregnancy and birth weight of infants.

**Methods:**

Systematic literature search was performed in Medline (via PubMed), Embase, Scopus, and CINAHL with no restrictions on language or time until May 2024. All observational studies that examined the relationship between maternal smokeless tobacco use and low birth weight of infants were eligible for inclusion. Methodological quality of included studies was assessed using the Newcastle Ottawa Scale.

**Results:**

Thirty-three studies were eligible for the review, including twenty-eight cohort, three case-control and two cross-sectional studies. A statistically significant association between use of smokeless tobacco and low birth weight was reported in thirteen studies. Eleven studies reported a statistically significant reduction in mean birth weight in maternal smokeless tobacco users. Pooled estimates of eighteen studies with 733,061 participants showed that there was a statistically significant association (OR = 2.25 [1.63, 3.11] P<0.001); between maternal smokeless tobacco use during pregnancy and low birth weight. Subgroup analysis found a significant association between mishri consumption during pregnancy and low birth weight (n = 646 participants, OR = 10.98 [2.03, 59.34], P = 0.005), but not betel nut (n = 8007 participants, OR = 1.02 [0.84, 1.25]), betel quid (n = 483 participants, OR = 1.51 [0.47, 4.89]) or khat (n = 475 participants, OR = 1.41 [0.64–3.09]).

**Conclusions:**

This review presents an association between maternal smokeless tobacco use and low birth weight, and reduction in mean birth weight. It is suggested that cessation and reduction of maternal smokeless tobacco use should receive specific attention within routine prenatal care.

**Implications:**

The results of this study highlight the need for further preventive public health campaigns to create awareness about detrimental effects of smokeless tobacco on foetal outcomes. Patient education in the primary care setting will aid in promoting smokeless tobacco cessation prior to pregnancy.

## Introduction

The prevalence of low birth weight infants in a population is an important indicator of complex public health issues including long term maternal malnutrition, ill health and poor antenatal health care. Of the 19.8 million low birthweight babies born in 2020, over 40% were born in South Asia [1]. World Health Organization (WHO) has defined low birth weight as weight at birth of <2500 gm (ICD-11 diagnosis code KA21.2) [2]. It is not only a predictor of neonatal mortality and morbidity, but also increases the risk of stunted growth, diminished cognitive development and chronic disease later in life [3–5]. Moreover, it is a primary outcome indicator in the core set of indicators for the Global Nutrition Monitoring Framework; and more than 90% of countries are off track to meet the year 2030 low birth weight target as set by UNICEF [3]. Low birth weight has a multi-factorial aetiology, with an increased risk associated with various maternal factors such as tobacco consumption, maternal weight, substance misuse, maternal age and deprivation, pregnancy interval and birth order of the child [6].

Although cigarette smoking is the most common form of tobacco use globally, smokeless tobacco is consumed by over 300 million people worldwide and is a growing public health concern. Geographically, >85% of the smokeless tobacco-related burden was in South and Southeast Asia, and in 2017 an estimated 2.5 million disability adjusted life years and 90,791 lives were lost worldwide due to cancers that can be attributed to smokeless tobacco [7]. Smokeless tobacco products can be found in many forms in different regions around the world, which vary in their preparation, method of use and components. In developed countries, common forms of consumption include snuff (finely ground dry or moist tobacco inhaled nasally) in Sweden, chewing tobacco (dried tobacco leaves) in the United States of America and pituri (chewed smokeless tobacco) amongst indigenous Australian populations. However, in developing countries, tobacco is usually chewed with other ingredients, such as betel (areca) nut, betel leaf, and lime [8]. Other forms of smokeless tobacco used in developing countries include mishri which is roasted ground tobacco and gutkha which is a mixture of tobacco with slaked lime, areca nut, acacia plant extract and spices.

There is increasing evidence to demonstrate that components of smokeless tobacco such as nicotine are toxicogenic and may increase risk of negative maternal-foetal outcomes if consumed during pregnancy [9,10]. Metabolism of nicotine in the human body gives rise to tobacco specific N-nitrosamines (TSNAs) which have been classified as a Group 1 carcinogen [11]. Moreover, nicotine can impair cell differentiation and induce a pro-inflammatory response that inhibits the agonistic action of retinoic acid, which is important for embryonic development [9]. Along with nicotine, there are up to 50 other harmful compounds found in tobacco which may impair foetal development by inducing both oxidative and endoplasmic reticulum stress responses in placental trophoblastic cells [10].

The use of smokeless tobacco is very common amongst women as it easily available, socially, culturally and ceremonially accepted; along with the misconception that it is less harmful as compared to smoking [12]. A study based on Demographic and Health Surveys data collected between 2010 and 2016 from 1,310,716 women across 42 low- and middle-income countries estimated that the prevalence of smokeless tobacco use was 0.56% (95%CI: 0.33–0.84) among pregnant women and 0.78% (95%CI: 0.35–1.37) among non-pregnant women of reproductive age, with particularly high prevalence in the South-East Asia region [13]. Moreover, a study examining Global Adult Tobacco Survey data in India from 2016–2017 estimated that the prevalence of smokeless tobacco use among pregnant women in India was 7.4% [14]. Another recent study based on data from National Family Health- 5 Survey in India reported that 2.2% of pregnant women used smokeless tobacco; with gutkha, paan masala/paan with tobacco and khaini being the most popular forms [15]. These estimates are both considerably higher than the aforementioned estimate for low- and middle-income countries.

Some forms of smokeless tobacco such as betel nut are chewed into a mass (known as a quid) and retained behind the lip in the buccal space for extended durations. Previous studies have shown that smokeless tobacco use may be associated with higher levels of nicotine entering the systemic circulation via this route as compared to smoking, resulting in more potent harmful effects [16,17].

Although there have been observational studies which investigate smokeless tobacco use and its implications on pregnancy and birth outcomes, the evidence is limited and findings vary [17,18]. It is necessary to synthesise this information to facilitate evidence-based practice when advising women about the risks of smokeless tobacco use. This systematic review and meta-analysis aims to investigate the currently available evidence on the association between maternal smokeless tobacco use during pregnancy and low birth weight infants, in comparison to those mothers who did not consume smokeless tobacco during pregnancy, and to assess and summarise the main features, methodological quality and results of the included studies from all across the globe with no limits on the time period of studies. This review is the first to perform sub-group analysis to explore any variation in effect of different types of smokeless tobacco on birth weight. Additionally, a previous review reported on the effects of non-combustible tobacco during pregnancy, however only included nicotine replacement therapies, snuff, iqmik and e-cigarettes [18]. This review includes a much wider range of smokeless tobacco types, including snuff, betel nut, gutka, iqmik, mishri, pituri and khat, with or without additives.

## Methods

This study is registered at the International Register of Prospective Systematic Reviews (PROSPERO) under the CRD number 42022381144. This review is reported according to Preferred Reporting Items for Systematic Reviews and Meta-Analyses (PRISMA) guidelines [19].

### Eligibility criteria

All observational studies (case-control, cohort and cross-sectional) that examined the relationship between maternal smokeless tobacco use and low birth weight of infants were included. Females in any healthcare setting were considered. Smokeless tobacco consumption of at least one form such as snuff, betel nut, gutka, iqmik, mishri, pituri and khat, with or without additives, used in any amount or frequency, during or at some time during pregnancy was considered. This review included only those data for participants with ongoing smokeless tobacco use during pregnancy and excluded former users who had quit prior to the pregnancy being evaluated in the study. Data from studies that included women who concurrently used both smokeless and smoking tobacco was excluded from this review. A total of 11 papers were excluded from this review for the following reasons: conference abstract (3 studies), effect of smokeless tobacco on birth weight not measured (7 studies), pregnant women exposed to other forms of tobacco as well as smokeless tobacco (1 study).

### Search methods

Four electronic databases (Medline (via PubMed), Embase, Scopus, CINAHL) were searched from inception to 31 May 2024 for studies published in peer-reviewed journals in any language. This included screening and reading titles, abstracts and full texts of papers, as well as excluding duplicates. The detailed search strategy, including search terms and Boolean operators used, can be found in Additional file 1. There were no date or language restrictions applied. Reference lists from the selected studies and grey literature were also hand searched, and authors were contacted for any unpublished studies.

Duplicate studies were excluded using Endnote X9. Subsequently, two reviewers (AM and BG) independently reviewed the titles and abstracts of studies. Any disagreements were settled by consensus between the authors. The full texts of all remaining studies were obtained and evaluated using predetermined inclusion and exclusion criteria.

### Data extraction

A standardised data sheet was prepared and used to extract the following data from the studies: title, first author’s name, year of publication, country of the study, time frame of study, study design, sample characteristics (sample size, number of cases/exposed and controls/unexposed), definition of smokeless tobacco and low birth weight, duration of follow up in cohort studies, adjustment for confounding, and the estimated risk with corresponding 95% confidence intervals (CI). The authors (AM and BG) independently performed the data extraction and resolved any doubts through consensus.

### Quality assessment

The methodological quality of included studies was assessed using the validated Newcastle-Ottawa Scale. This is a risk of bias assessment tool for observational studies that is recommended by the Cochrane Collaboration [20]. This scale uses a ‘star system’ and includes eight items clustered into three broad domains: the selection of the study groups (representativeness of the exposed cohort, selection of non-exposed cohort and ascertainment of exposure, demonstration that outcome of interest was not present at start of study); comparability (comparability of cohorts on the basis of the design or analysis controlled for confounders); and outcome (assessment of outcome, and whether follow-up was long enough for outcomes to occur and adequacy of follow-up of cohorts). One point can be assigned for each item if the study methods have been explained appropriately with a total maximum possible score of nine. Comparability is the only category which can score up to two points (one if the most important confounders have been adjusted for in the analysis and a second star if any other adjustments were made) [21]. Scores on the Newcastle-Ottawa scales can be converted to AHRQ standards (good, fair, and poor) based on the following thresholds: good quality if 3 or 4 stars in selection domain and 1 or 2 stars in comparability domain and 2 or 3 stars in outcome/exposure domain; fair quality if 2 stars in selection domain and 1 or 2 stars in comparability domain and 2 or 3 stars in outcome/exposure domain; poor quality if 0 or 1 star in selection domain or 0 stars in comparability domain or 0 or 1 stars in outcome/exposure domain [21].

### Data synthesis

Each study was individually analysed by tabulating the study characteristics and comparing against the planned groups for each synthesis to find quantitative measures of effect for the exposure and outcome. The effects of measure used for dichotomous data were odds ratio (OR) and relative risk (RR) along with their corresponding 95% CI. The OR was calculated by the authors (AM and BG) when it was not included in the published study, but adequate data was provided for calculation. Odds ratios were calculated using the MedCalc online calculator (https://www.medcalc.org/calc/odds_ratio.php). All statistical analyses, including meta-analysis of studies examining the association between low birth weight and maternal smokeless tobacco use, were performed using Review Manager (RevMan) version 5.3 [22]. Data from studies with the same outcome (low birth weight defined as <2500grams) was pooled using forest plots in meta-analysis. A random effects model for heterogeneity was employed which generates results that generalize to a range of populations and study designs, with a p value less than 0.05 considered as significant. Heterogeneity between studies was evaluated for each analysis using Cochran’s Q statistic (measure of weighted square deviations), with N-1 degrees of freedom (where N is the number of studies), between studies variance (T2), and ratio of the true heterogeneity to total observed variation (*I*^2^). By calculating *I*^2^ values, an *I*^2^ less than 25% was deemed to have low heterogeneity, 25–75% to have medium heterogeneity, and greater than 75% to have high heterogeneity. Possible causes for heterogeneity were investigated through sensitivity analysis, which excluded those studies which did not adjust for confounding factors. The data points for the random effects meta-analysis were made up of the logarithms of the effect sizes and their corresponding standard errors. Subgroup analysis was performed based on the type of smokeless tobacco consumed by study participants during pregnancy (betel nut, khat, mishri, betel quid).

Publication bias was assessed through visual inspection of the symmetry of funnel plots, which were constructed by plotting the logarithmically transformed ORs against the standard error of the associated log OR. Since the confounding variables used in the multivariate regression model varied significantly between studies, unadjusted effect estimates were used in the meta-analysis, which were calculated using RevMan.

## Results

**Fig 1:**
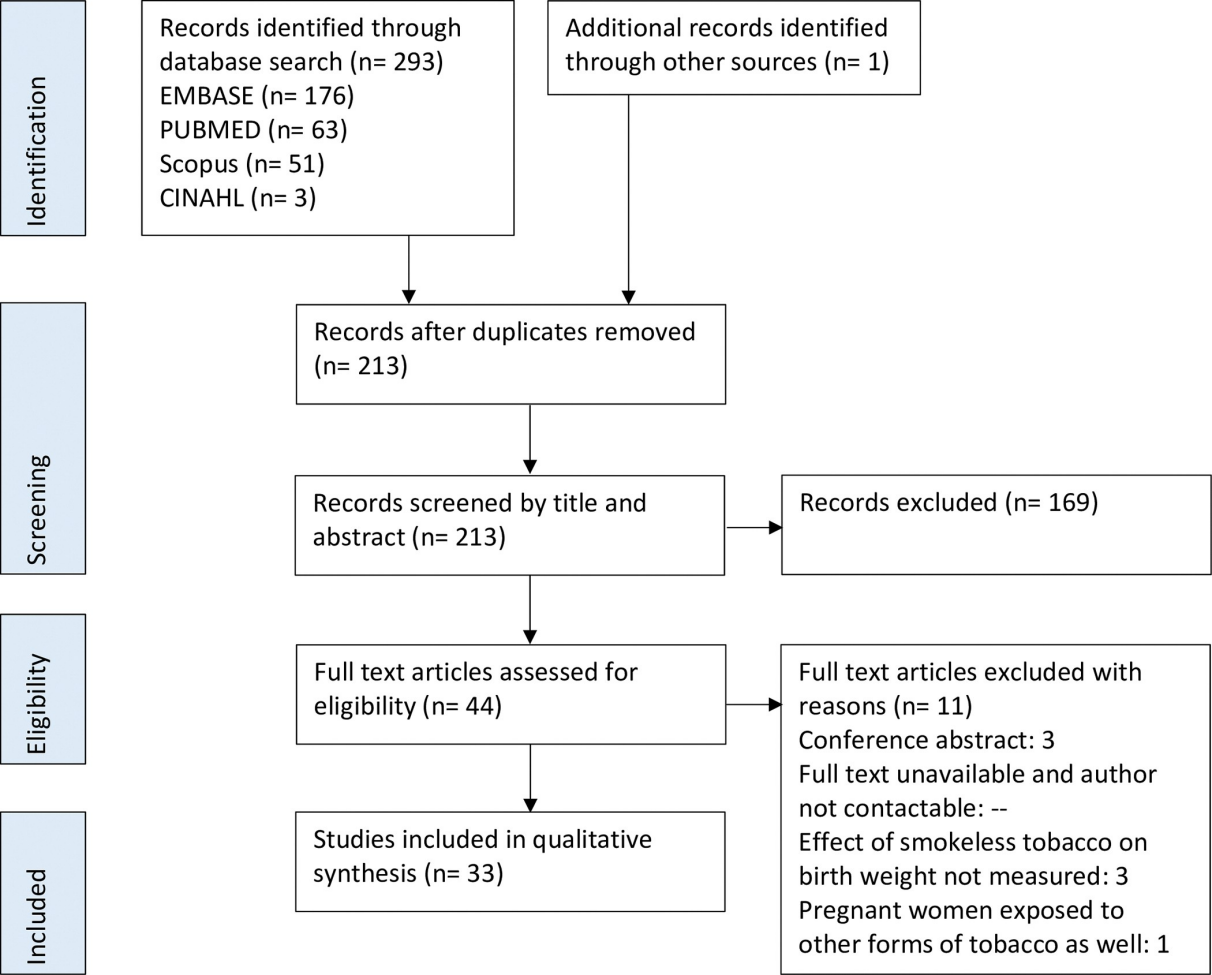
Flowchart showing process of literature search according to Preferred Reporting Items for Systematic Review and Meta-Analysis (PRISMA) guidelines.

The search yielded a total of 294 studies (176 from EMBASE, 63 from PUBMED, 51 from Scopus, 3 from CINAHL and 1 through hand search of reference lists). After duplicates were removed and the title and abstract of studies were reviewed; 44 studies were considered potentially eligible for this review and assessed as full-texts based on predetermined inclusion and exclusion criteria. There were 11 articles excluded from this review, and 33 articles included, further details of which are shown in Fig 1. Despite having no limitation on language of publication, all eligible studies found through systematic search of the literature were published in English.

The characteristics of the 33 studies included in this review are presented in Table 1 which describes the location, sample size, definitions of outcome, type of smokeless tobacco, and quantitative measures of outcome. There were eighteen studies from Asia (ten from India, three from Yemen, two from Taiwan, one each from Pakistan, Thailand and Indonesia), six studies from Europe (five from Sweden and one from Sweden and Norway), four studies from Oceania (two from Papua New Guinea, one from Palau and one from Australia), four studies from Africa (three from Ethiopia and one from South Africa), and one study from the United States of America. This review included a total sample size of 2,996,627 pregnant women. The largest study was from Sweden with a sample size of 1,070,013 women [23], contrary to the smallest study being reported from Australia with a sample size of 50 pregnant women [24]. The predominant form of smokeless tobacco used in Ethiopian and Yemen studies was khat; on the other hand, in countries from Asia and Oceania it was betel/areca nut; and in India it was mishri. Interestingly, snuff was used by both the European and South African subjects, in comparison to iqmik and pituri used by subjects in the study from USA and Australia respectively. None of the studies reported a quantified measure of exposure, and most did not quantify frequency of smokeless tobacco use.

**Table 1:** Characteristics of studies which explored the association between maternal smokeless tobacco use during pregnancy and birth weight.

Reference	Region	Time frame	Study design	Definition of birth weight outcome	Smokeless tobacco type	Definition of exposure	Sample population	Number of exposed	Number of non-exposed	Odds ratio and 95% CI for low birth weight
(Abdul Ghani et al, 1987) [25]	Yemen Arab Republic	1984	Cohort	Mean birth weight (g)	Khat	Self-reported use of khat during pregnancy	830	414	295	Not available
(Aziz et al, 2021) [26]	Pakistan	2013–2019	Cohort	Low birth weight (<2500g)	Gutkha	Self-reported use of gutkha during pregnancy	702	505	197	Not available
(Baba et al, 2013) [27]	Sweden	1999–2010	Cohort	Small for gestational age (birthweight > 2 standard deviations below the mean weight for gestational age)	Snuff	Self-reported use of snuff 3 months before pregnancy	846411	23514	663649	1.26 (1.09–1.46)
(Berger et al, 2016) [28]	Palau	2007–2013	Cohort	Low birth weight (<2500g)	Betel nut	Self-reported chewing of betel nut with tobacco during pregnancy	1171	893	278	2.4 (1.0–6.0)
(Charlette et al, 2022) [29]	India	2021	Cohort	Low birth weight (<2500g)	Unspecified	Self-reported use of smokeless tobacco during pregnancy	463	23	440	2.58 (1.1–6.06)
(Chue et al, 2012) [30]	Thailand	1997–2006	Cohort	Low birth weight (<2500g)	Betel nut	Self-reported use of areca nut during pregnancy	7685	4963	2722	0.9091 (0.7694–1.0742)
(de Costa and Griew, 1982) [31]	Papua New Guinea	1981	Cohort	Low birth weight (<2500g)	Betel nut	Self-reported use of betel nut throughout pregnancy	800	400	400	1.4227 (0.418–2.4042)
(Demelash et al, 2015) [32]	Ethiopia	2013	Case-control	Low birth weight (<2500g)	Khat	Self-reported use of khat during pregnancy	387	48	339	6.4 (2.42–17.10)
(Dendir and Deyessa, 2017) [33]	Ethiopia	2014	Case-control	Low birth weight (<2500g)	Khat	Self-reported use of khat during pregnancy	347	55	291	2.83 (1.35, 5.93)
(England et al, 2012) [34]	USA	1997–2005	Cohort	Low birth weight (<2500g)	Iqmik	Self-reported use of iqmik or commercial chew during pregnancy	358	237	121	Not available
(England et al, 2003) [35]	Sweden	1999–2000	Cohort	small for gestational age (birthweight > 2 standard deviations below the mean weight for gestational age)	Snuff	Self-reported use of snuff during pregnancy	12284	789	11495	1.25 (0.72–2.17)
(Eriksson et al, 1991) [36]	Yemen Arab Republic	1991	Cohort	Low birth weight (<2500g)	Khat	Self-reported use of khat during pregnancy	1141	142	382	1.3302 (0.8495 to 2.0831)
(Ganganahalli et al, 2017) [37]	India	2011	Cohort	Low birth weight (<2500g)	Mishri	Self-reported use of mishri during pregnancy-	516	258	258	Not available
(Gupta and Subramoney, 2004) [38]	India	2002	Cohort	Low birth weight (<2500g)	Unspecified	Self-reported use of a smokeless tobacco product at least once a day for the past six months during pregnancy	1217	206	768	Not available
(Idris et al, 2020) [39]	Yemen	2016	Case-control	Low birth weight (<2500g)	Khat	Self-reported use of khat during pregnancy	252	83	43	1.76 (1.06–2.92)
(Juarez and Merlo, 2013) [40]	Sweden	2002–2010	Cohort	Mean birthweight (g)	Snuff	Self-reported use of snuff during pregnancy	938932	8339	591690	Not available
(Kreyberg et al, 2019) [41]	Norway and Sweden	2014–2016	Cohort	Mean birthweight (g)	Snuff	Self-reported use of snuff during pregnancy	2313	150	2046	Not available
(Krishna, 1978) [42]	India	1971–1972	Cohort	Mean birthweight (g)	Unspecified	Self-reported use of oral tobacco (a wad of locally grown and cured tobacco kept in the mouth) for 8 to 10 hours per day during pregnancy	1357	209	1148	Not available
(Krishnamurthy and Joshi, 1993) [43]	India	1993	Cohort	Low birth weight (<2500g)	Unspecified	Self-reported use of smokeless tobacco, mostly mishri, during pregnancy	178	42	41	3.2(1.5–6.9)
(Madley-Dowd et al, 2021) [23]	Sweden	1999–2010	Cohort	Small for gestational age (birthweight > two standard deviations below the mean weight for gestational age according to the gender-specific Swedish fetal growth curves)	Snuff	Self-reported use of snuff during pregnancy	1070013	14665	1055348	1.05(0.94–1.17)
(Mallick, 2021) [44]	India	2015–2016	Cohort	Low birth weight (<2500g)	Unspecified	Self-reported use of chewing tobacco during pregnancy	57582	3629	53953	1.163 (1.058–1.227]
(Ome-Kaius et al, 2015) [45]	Papua New Guinea	2009–2013	Cohort	Low birth weight (<2500g)	Betel nut	Self-reported use of betel nut during pregnancy	2700	1459	310	0.94 (0.65–1.38)
(Pratinidhi et al, 2010) [46]	India	2003–2005	Cohort	Low birth weight (<2500g)	Mishri	Self-reported use of mishri during pregnancy	705	218	487	2.4026 (1.5206–3.7963)
(Pratinidhi et al, 2014) [47]	India	2011	Cohort	Low birth weight (<2500g)	Mishri	Self-reported use of mishri during pregnancy	516	258	258	67.9016 (37.3926–123.3033)
(Ratsch et al, 2022) [24]	Australia	2021	Cohort	Mean birthweight (g)	Pituri	Self-reported use of pituri during pregnancy	50	19	31	1.75 (0.3152–9.7161)
(Rygh et al, 2019) [48]	Norway	2012–2017	Cohort	Mean birthweight (g)	Snuff	Self-reported use of snuff during the third trimester of pregnancy	19767	201	9213	Not available
(Senn et al, 2009) [49]	India	2007–2008	Cross-sectional	Low birth weight (<2500g)	Betel nut	Self-reported use of betel nut chewing during pregnancy	310	292	18	1.9 (0.4–17)
(Steyn et al, 2006) [50]	South Africa	1990	Cohort	Mean birthweight (g)	Snuff	Self-reported use of snuff during pregnancy	1593	120	1376	Not available
(Sulistiyani et al, 2019) [51]	Indonesia	2018	Cross-sectional	Low birth weight (<2500g)	Unspecified	Self-reported use of smokeless tobacco during pregnancy	99	18	81	Not available
(Verma et al, 1983) [52]	India	1978–1979	Cohort	Mean birthweight (g)	Unspecified	Self-reported use of chewing tobacco during pregnancy, minimum 150 mg/day	140	70	70	Not available
(Wang and Lee, 2012) [53]	Taiwan	2005	Cohort	Low birth weight (<2500g)	Betel nut	Self-reported use of betel quid during pregnancy	8432	19	8413	0.368 (0.048–2.841)
(Wondemagegn et al, 2024) [54]	Ethiopia	2022	Cohort	Small for gestational age at birth was declared (birth weight <10th percentile of the sex specific birth weight for gestational age reference curve)	Khat	Self-reported use of khat during pregnancy	344	156	164	Not available
(Yang et al, 2008) [55]	Taiwan	2003–2004	Cohort	Low birth weight (<2500g)	Betel nut	Self-reported use of betel quid during pregnancy	1264	464	800	2.40 (1.21–4.80)

There was some variation in the methodological quality of included studies as summarised in Table 2, although most studies were of good quality as per the Newcastle-Ottawa Scale for Quality Assessment. All of the studies had an overall score of 6 or more.

**Table 2:** Quality assessment of included studies as per the Newcastle-Ottawa scale.

Study	Selection				Comparability of cohorts on the basis of the design or analysis controlled for confounders	Outcome			Overall score (maximum 9)
	Representativeness of the exposed cohort	Selection of non-exposed cohort	Ascertainment of exposure	Demonstration that outcome of interest was not present at start of study		Assessment	Duration of follow up (adequate follow up minimum 9 months for outcome to occur)	Adequacy of follow up (>80%)	
(Ghani et al, 1987) [25]	1	1	1	1	0	1	1	1	7
(Aziz et al, 2021) [26]	1	1	1	1	2	1	1	1	9
(Baba et al, 2013) [27]	1	1	1	1	2	1	1	1	9
(Berger et al, 2016) [28]	1	1	1	1	2	1	1	1	9
(Chue et al, 2012) [30]	0	1	1	1	0	1	1	1	6
(Charlette et al, 2022) [29]	1	1	1	1	1	1	1	1	8
(de Costa and Griew, 1982) [31]	1	1	1	1	1	1	1	1	8
(Demelash et al, 2015) [32]	1	1	1	1	1	1	1	1	8
(Dendir and Deyessa, 2017) [33]	1	1	1	1	1	1	1	1	8
(England et al, 2012) [34]	1	1	1	1	2	1	1	1	9
(England et al, 2003) [35]	1	1	1	1	2	1	1	1	9
(Eriksson et al, 1991) [36]	1	1	0	1	0	1	1	1	6
(Ganganahalli et al, 2017) [37]	1	1	1	1	2	1	1	1	9
(Gupta and Subramoney, 2004) [38]	1	1	1	1	2	1	1	1	9
(Idris et al, 2020) [39]	1	1	1	1	1	1	1	1	8
(Juarez and Merlo, 2013) [40]	1	1	1	1	2	1	1	1	9
(Kreyberg et al, 2019) [41]	1	1	0	1	2	1	1	1	8
(Krishna, 1978) [42]	1	1	1	1	0	1	1	1	7
(Krishnamurthy and Joshi, 1993) [43]	1	1	1	1	0	0	1	1	6
(Madley-Dowd et al, 2021) [23]	1	1	1	1	2	1	1	1	9
(Mallick, 2021) [44]	1	1	1	1	2	1	1	1	9
(Ome-Kaius et al, 2015) [45]	1	1	0	1	1	1	1	0	6
(Pratinidhi et al, 2010) [46]	1	1	1	1	0	1	1	1	7
(Pratinidhi et al, 2014) [47]	1	1	1	1	2	1	1	1	9
(Ratsch et al, 2022) [24]	0	1	1	1	1	1	1	1	7
(Rygh et al, 2019) [48]	1	1	1	1	2	1	1	1	9
(Senn et al, 2009) [49]	1	1	1	1	1	1	1	1	8
(Steyn et al, 2006) [50]	1	1	1	1	2	1	1	1	9
(Sulistiyani et al, 2019) [51]	1	1	1	1	0	1	1	0	6
(Verma et al, 1983) [52]	1	1	1	1	2	1	1	1	9
(Wang and Lee, 2012) [53]	1	1	1	1	2	1	1	1	9
(Wondemagegn et al, 2024) [54]	1	1	1	1	1	1	1	1	8
(Yang et al, 2008) [55]	1	1	1	1	2	1	1	1	9

### Effects of exposure

Thirty-three studies included in this review were clinically and methodologically diverse. There were twenty-eight cohort studies, three case-control and two cross-sectional studies included in this systematic review. Crude odds ratios were presented in only twelve [23,29,32,33,35,38,39,43–45,49,53,55] of the included studies, and could be calculated by the authors using the data published for a further six studies [24,30,31,36,46,47]. Relative risk was presented in three of the included studies [26,38,54]. A statistically significant association between use of smokeless tobacco and low birth weight was reported in thirteen of the studies [27–29,32,33,37–39,43,44,46,47,55]. All of these studies defined low birth weight as <2500 grams. Mothers who used smokeless tobacco during pregnancy had up to almost 68 times (67.90 [37.39–123.30]) greater risk for low birth weight infants as reported in the study conducted in India in 2014 by Pratinidhi et al [47]. On the contrary, Wang and Lee reported non-significant results (0.37 [0.05–2.84]) for a study conducted on 8432 women in Taiwan [53].

A statistically significant reduction in mean birth weight was reported in maternal smokeless tobacco users in a further eleven studies [25,27,30,31,38,46–49,52,55]. However, four studies reported that there was no statistically significant difference in birth weight in infants born to mothers who used smokeless tobacco during pregnancy [24,34,41,50].

Out of all the studies included in this review, seven studies reported on snuff users [23,27,35,40,41,48,50], seven reported on betel nut users [28,30,31,45,49,53,55], six reported on khat users [25,32,33,36,39,54], three on mishri [37,46,47], one reported on gutka [26], iqmik [34] and pituri [24] users respectively, and seven did not specify the type of smokeless tobacco used by participants [29,38,42–44,51,52].

Three studies on khat [32,33,39], two on mishri [46,47], one on snuff [27], one on betel quid [55] and none on areca nut, betel nut, pituri, iqmik or gutka reported a statistically significant association between maternal smokeless tobacco use and low birth weight. One study on khat [25], three on snuff [35,40,48], one on mishri [37], two on betel nut [49,55] and none on iqmik or pituri reported a significant reduction in mean birthweight in infants born to mothers who used smokeless tobacco during pregnancy.

### Meta-analysis

**Fig 2:**
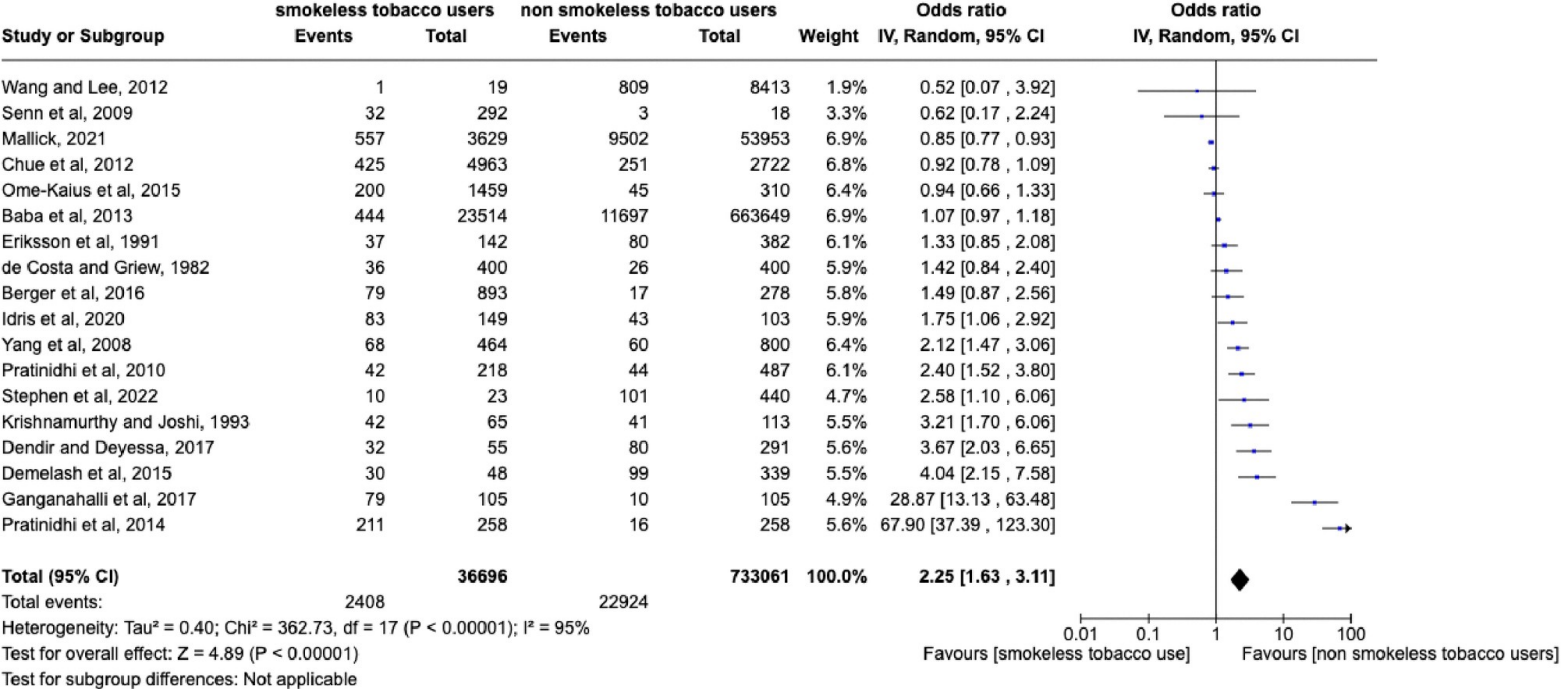
Forest plot for effect of maternal smokeless tobacco consumption compared with no tobacco consumption during pregnancy on risk of low birth weight in infants.

Eighteen studies involving 733,061 participants were included in the meta-analysis, which is shown in Fig 2 [27–33,36,37,39,43–47,49,53,55]. Other studies were not included due to the inadequate reporting of measures of effect size such as OR or RR and provision of insufficient data for these values to be calculated by the authors of this review. The probability of low birth weight infants was greater in those women who consumed smokeless tobacco during pregnancy as compared to those who did not consume any tobacco products during pregnancy. The overall pooled estimate under the random effects model showed that there was a statistically significant association (OR = 2.25 [1.63, 3.11], P = 0.005), between maternal smokeless tobacco use during pregnancy and low birth weight. Smokeless tobacco users were more than twice as likely to have low birth weight infants as compared to non-tobacco users. The test for heterogeneity produced Tau square of 0.40, I^2^ =  95%, test for overall effect z  =  4.89, (P<0.001). This demonstrates a high level of heterogeneity between studies. The highest risk estimates observed were OR = 67.90 (37.39, 123.30), in a study conducted in India, which evaluated the effects of maternal mishri consumption during pregnancy on birth weight [47]. However, the wide 95% CIs suggest that this effect estimate may be due to factors such as the small sample size of the study which decreases the reliability of results.

**Fig 3:**
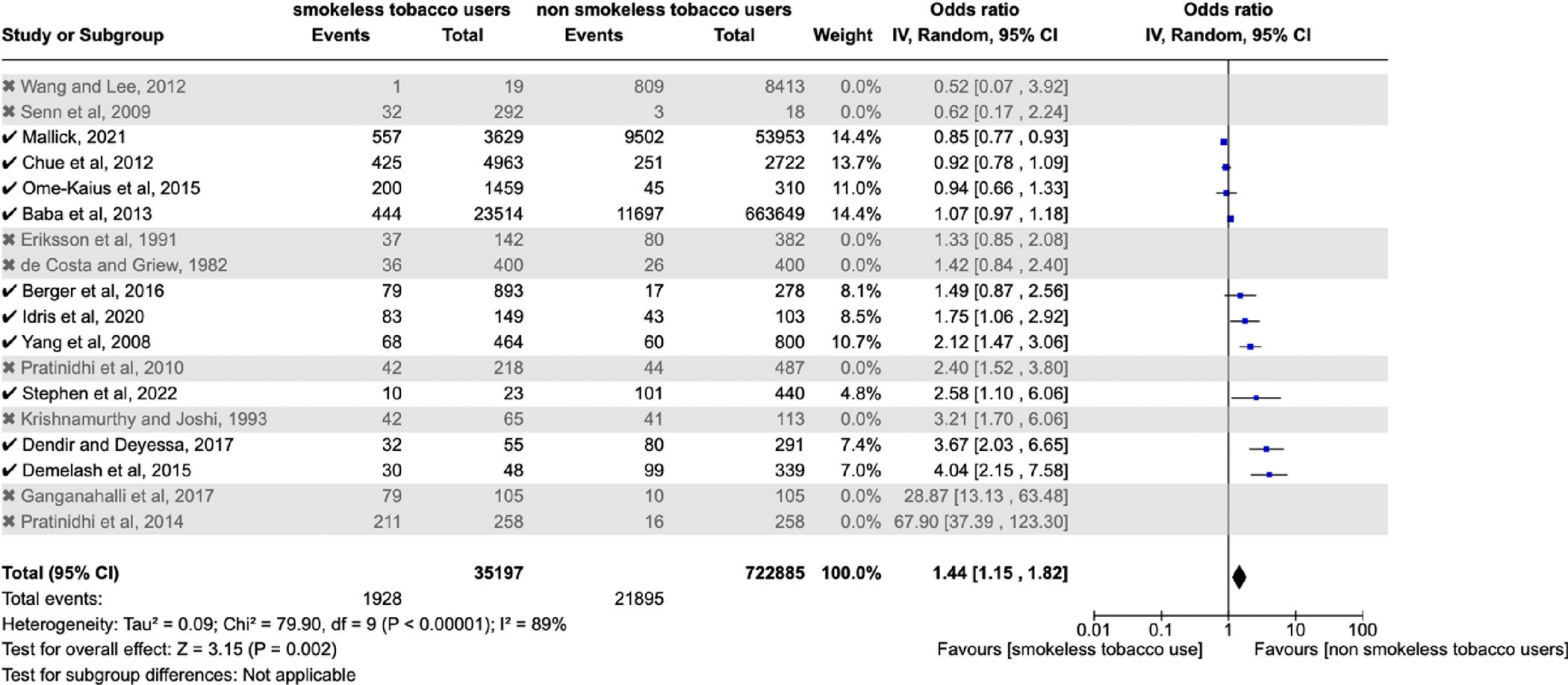
Sensitivity analysis for effect of maternal smokeless tobacco consumption compared with no tobacco consumption during pregnancy on risk of low birth weight in infants.

In order to further investigate heterogeneity, sensitivity analysis was performed which excluded eight studies that did not adjust for confounding factors [31,36,37,43,46,47,49,53]. When the meta-analysis was performed without these studies, the pooled estimate of effect size was an OR of 1.44 (1.15–1.82), P = 0.002, as shown in Fig 3. However, the heterogeneity in the sensitivity analysis remained high as I^2^ = 89%, as compared to an I^2^ value of 95% in the meta-analysis which did not exclude any studies. Therefore, the aforementioned eight studies were not excluded from the meta-analysis. The results of the review should be interpreted with caution based on this sensitivity analysis. Nevertheless, the findings of this paper generate hypotheses for further investigations and future research.

**Fig 4:**
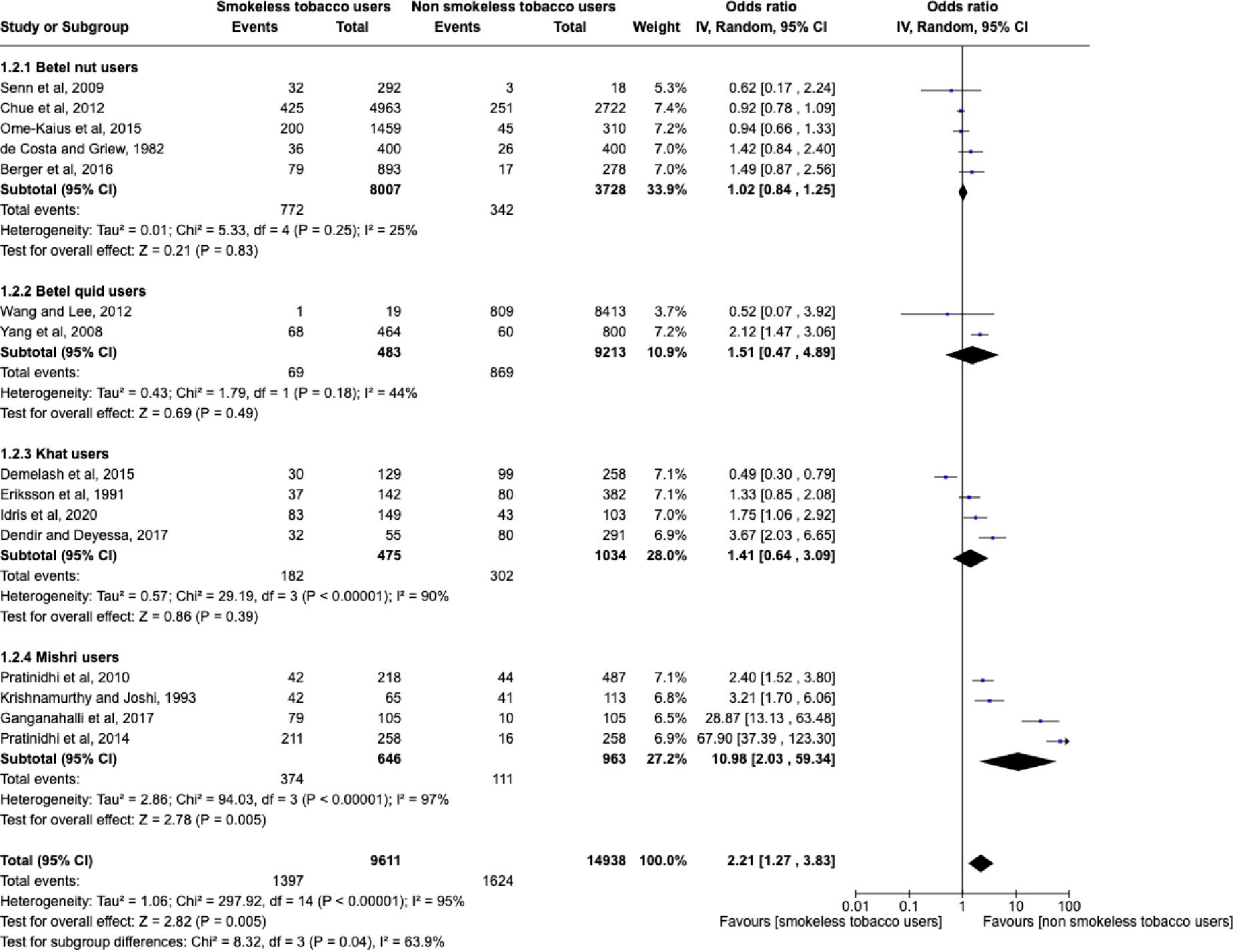
Subgroup analyses of the effect of maternal smokeless tobacco consumption compared with no tobacco consumption during pregnancy on risk of low birth weight in infants.

Subgroup analysis was performed for five studies for betel nut [28,30,31,45,49], two studies for betel quid [53,55], four studies for khat [32,33,36,39], and four studies for mishri users, as shown in Fig 4 [37,43,46,47]. Subgroup analysis could not be performed for snuff users [23,27,35,40,41,48,50] as only one study provided sufficient data on snuff use and low birth weight to be included in the meta- analysis [27]. The results of subgroup analysis varied depending upon the type of smokeless tobacco use and we found nearly eleven times greater probability of low birth weight in infants of mothers who consumed mishri during pregnancy (OR = 10.98 [2.03, 59.34], P = 0.005). However, no statistically significant association with low birth weight was found for betel nut (OR = 1.02 [0.84, 1.25], P = 0.83), betel quid (OR = 1.51 [0.47, 4.89], P = 0.49) or khat (OR = 1.41 [0.64, 3.09], P = 0.39) users. This may be due to inadequate pooled sample size of the included studies and the influence of confounding factors.

**Fig 5:**
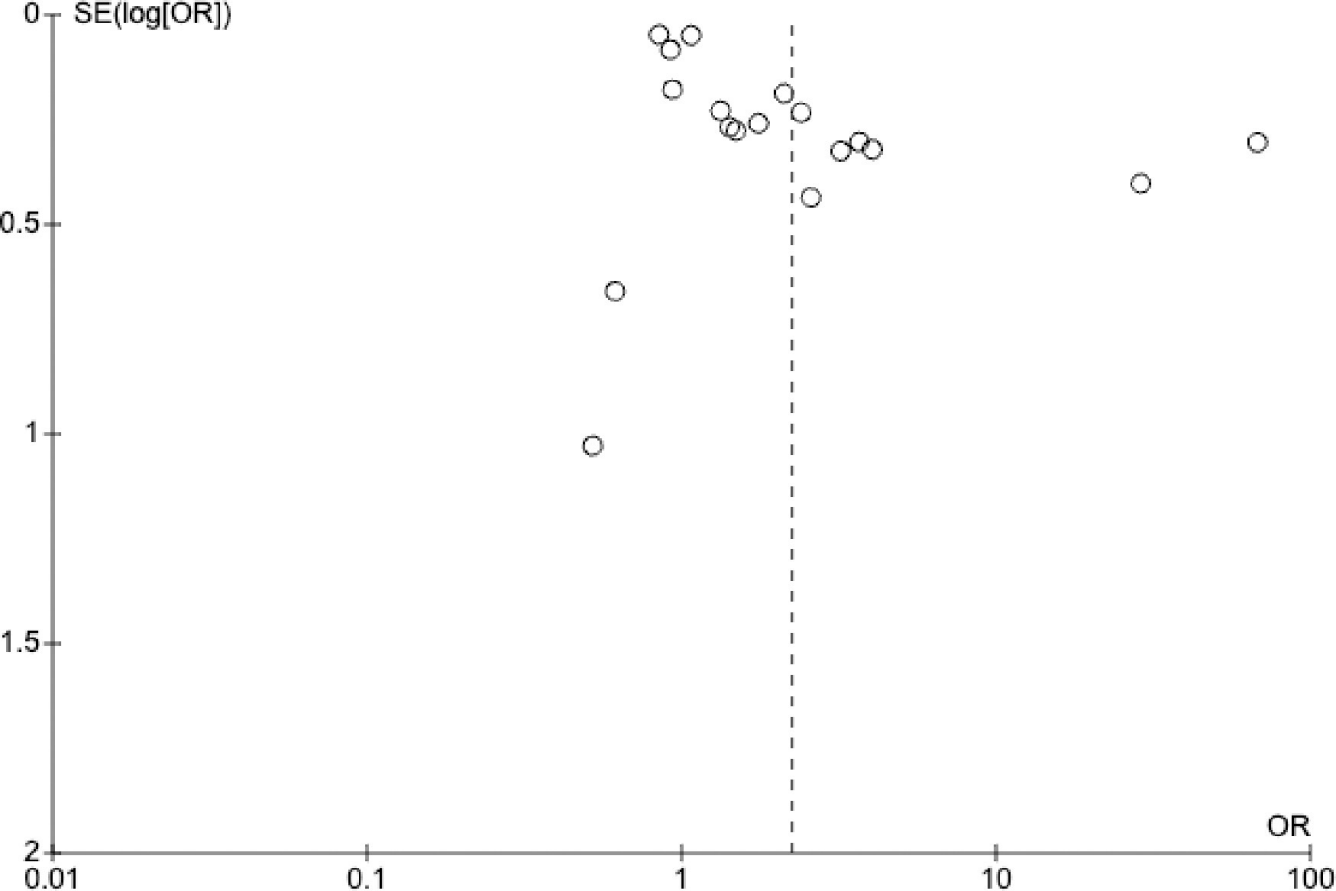
Funnel plot of all studies included in this meta-analysis.

**Fig 6:**
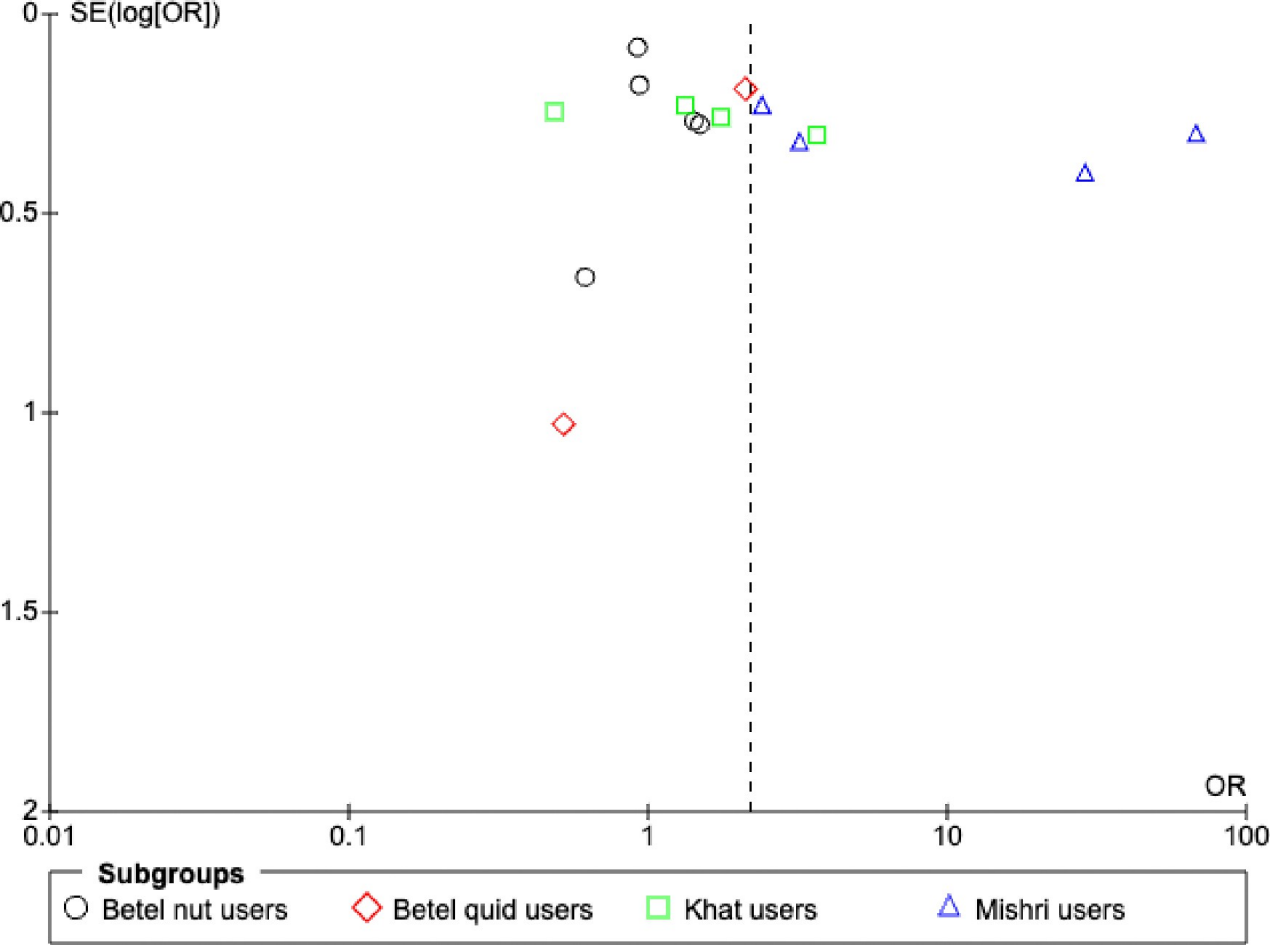
Funnel plot of studies included in the subgroup analysis based on type of smokeless tobacco consumed during pregnancy.

Most of the studies are aggregated towards the centre of the funnel plot (see Figs 5 and 6), which is symmetrical on visual inspection, indicating that there was no publication bias in this meta-analysis.

## Discussion

In this systematic review and meta-analysis, we aimed to quantify the effect of maternal smokeless tobacco use during pregnancy on low birth weight of infants and our review suggests that there is a significant association between the same. These results may not present the true picture and may be underestimated due to the existence of many potential confounding factors such as the maternal age, socio-economic background, other substance abuse, malnutrition, genetics, preterm birth, environmental factors like housing, and lack of access to adequate health care services. Additionally, countries which do not record the birthweight in national data sources have missing or incomplete information on newborns’ birthweight.

Though most of the studies were of good quality, they differed in frequency, duration, ingredient composition and administration practices of smokeless tobacco use. Different types of smokeless tobacco may contain varying levels of nicotine and additives, adding to the heterogeneity of the data studied. In fact, some forms of smokeless tobacco included in this review pose unknown and apparently higher risk as tobacco is a minor ingredient and these smokeless tobacco products primarily contain slaked lime, ash or other plant matter or spices [56].

This review found that there is a significant association between maternal smokeless tobacco use and low birth weight of infants, as well as reduction in mean birth weight. These findings are in agreement with the previous review conducted by England et al in 2010 who found that smokeless tobacco use in pregnant women was associated with a significant reduction in birth weight in both low income and high-income countries [57]. Similarly, Ratsch and Bogossian’s 2014 review [57] also reported that smokeless tobacco was associated with a reduction in mean birth weight as well as low birth weight across 21 observational studies. A significant association between smokeless tobacco use during pregnancy and low birth weight was found in 5 out of 7 studies included in the systematic review by Inamdar et al in 2014, and similarly to our review, they also reported high levels of heterogeneity in the results [58]. Our results are also in agreement with a meta-analysis conducted by Suliankatchi and Sinha in 2016 which found that smokeless tobacco use was significantly associated with increased risk of low birth weight (OR 1.88 [1.29–2.83]) [59]. Nonetheless, there are no previous reviews which conducted subgroup analysis to quantify any variations in this association across different types of smokeless tobacco.

Although there are few published studies on the prevalence of smokeless tobacco use in pregnant women, data suggests that use in these populations is generally higher in low income countries and in vulnerable communities within high income countries [60]. Additionally, previous cross-sectional studies conducted in India have demonstrated an inverse relationship between smokeless tobacco use among women, and level of education and income [13,18]. For instance, traditional forms of smokeless tobacco are used by Indigenous populations such as use of pituri in Australia [24,61] and iqmik by Alaska natives in the USA [34]. There is supporting evidence that pituri has nicotine content as high as 11mg/g and the addition of highly alkaline wood ash further raises its pH [62,63]. However, there is little research on these forms of smokeless tobacco and minority populations are also often under-represented in research studies, leading to a double-edged sword placing these populations at increased risk of poor maternal and foetal outcomes.

There are many pathways through which nicotine consumed via smokeless tobacco during pregnancy may influence birth weight of infants. Nicotine in the bloodstream produces both generic reproductive and pregnancy-specific biphasic responses, transitioning from initial simulation to depression in a dose-dependent and cumulative manner [64]. nicotine can transit from the systemic circulation into the placenta, and this has been evidenced through studies which found higher nicotine ratios in the umbilical venous and arterial cord bloods than in maternal serum [65] as well as nicotinic acetylcholine receptor (nAChR) binding in the developing and immature foetus [66]. This suggests that nicotine may exert its effects through both maternal and foetal biochemical pathways. There are varying levels of nicotine in different smokeless tobacco products, which suggests that some may pose greater risk. Studies have found that chewing tobacco products contained about 3.4 to 39.7mg/g of nicotine, while snuff products contained 4.7 to 24.8mg/g [67].

Studies conducted in India report a 100-200gm reduction in birth weight and an excess of preterm deliveries among Indian women who chewed tobacco during their pregnancy as compared to non-tobacco users [51,68]. Moreover, studies reported that Indian women who used mishri during their pregnancy had nearly three times greater risk of getting low birth weight babies [43,46].

Khat, which is chewed for its euphoric and stimulant properties, remains underexplored and the mechanism behind its effects on birth-weight is not known. However, one of the active substances in khat, d-norpseudoephedrine, has been shown to adversely affect the placental blood circulation. Khat also has an anorectic effect, which could lead to reduction in food intake by the pregnant woman and thus contribute to low birth weight [25,69].

Maternal betel and areca nut chewing may exert its harmful effects through similar mechanisms. Animal models have shown that prenatal areca nut chewing has embryotoxic and teratogenic effects [70]. Meanwhile a human study found that it is associated with focal inflammatory changes in the placental amniochorial membranes as well as decreased median diameter of blood vessels supplying placental villi on both the maternal and foetal surfaces [71].

Findings of this review have wider clinical and public health implications. Public health campaigns to increase awareness about the negative effects of smokeless tobacco in women of childbearing age, as well as education of both males and females by healthcare workers in the primary care setting will aid in promoting smokeless tobacco cessation prior to pregnancy. Scientific literature suggests that various forms of smokeless tobacco cessation programs including behavioural interventions, quit lines and pharmacotherapy such as varenicline and nicotine lozenges are cost-effective and highly efficacious in aiding patients to quit smoking [72]. Online support groups within a community can be set up to express solidarity and conviviality. Doctors, midwives and nurses can encourage early tobacco use cessation and educate patients about the potential harms to the foetus. It may also be beneficial to provide additional monitoring in the form of ultrasounds and/or antenatal clinic appointments to mothers who use smokeless tobacco to detect infants who may be small for gestational age and at risk of low birth weight. Such infants may also need to be monitored more closely after birth, which poses an added cost to the healthcare system which is already burdened in many countries where smokeless tobacco use has high prevalence, particularly in South Asia.

This review reveals the importance of screening for smokeless tobacco use during antenatal appointments, and subsequently counselling women to cease this during pregnancy as well as referring them to appropriate cessation services. This is not currently part of routine antenatal care; but should be considered especially in countries with high prevalence of smokeless tobacco use. However, such interventions may be challenging, as previous studies have found that engagement with smoking cessation services, particularly among pregnant women from deprived backgrounds is low despite initial interest in smoking cessation services. For instance, a study in England reported that although almost half of pregnant smokers were interested in cessation support, only 38% discussed quitting with a health professional and 27% actually accessed NHS cessation support [73]. This may be due to social stigma or lack of health literacy, and hence public health awareness campaigns should be implemented to increase health literacy surrounding the harms of smokeless tobacco consumption during pregnancy, through easily accessible channels such as social media or television or integration of pamphlets and posters at antenatal and general practice clinics. Although there has been limited research on smokeless tobacco cessation, a systematic review found that behavioural interventions such as clinician advice and telephone counselling via quitlines have high efficacy; while other interventions such as regular telephone support and pharmacological modalities like nicotine lozenges and varenicline were also beneficial [72]. Novel interventions for tobacco cessation such as narrative and picture-based digital storytelling delivered via text-messages may be promising, but these require further research trials to evaluate their efficacy [74]. It is therefore important that healthcare professionals are appropriately trained to provide smokeless tobacco cessation counselling, and school-based prevention and cessation programmes should be developed further.

Further research and quality observational studies are needed to strengthen this evidence base. None of the studies reported a quantified measure of exposure, most did not quantify frequency of smokeless tobacco use, and there are no studies which compare the effects of different forms of smokeless tobacco use. Consequently, a dose-response relationship could not be evaluated. It would be beneficial to include objective, quantitative measures of maternal tobacco consumption such as maternal and neonatal urinary and blood cotinine levels in further studies to better evaluate this association and establish the presence of dose response relationship [75].

### Limitations

Self-reported data regarding the use of smokeless tobacco during pregnancy was collected via interviews and hospital records in all the included studies. Consequently, there is a potential risk of recall bias in these studies since none of the studies reported any biochemical/objective measurements that were performed to ascertain tobacco use. Moreover, only sixteen [23,26–28,30,32,34,35,38–41,44,45,50,55] studies included in this review adjusted for confounding factors which may affect the reliability of these results given that it is established that many biopsychosocial factors such as maternal diet and environment can influence birth weight.

Many studies did not evaluate for and exclude those participants who consumed both smoking and smokeless tobacco, which may be an additional confounding variable. Smokeless tobacco use in some of the included studies was concurrent with cigarette smoking and other risk factors such as alcohol consumption, second hand or household smoke exposure and malnutrition [76]; hence it is challenging to ascertain whether differences in birth weight are attributable to the smokeless tobacco use itself. Additionally, the included studies did not evaluate whether participants engaged in concurrent use of multiple different types of smokeless tobacco during pregnancy, and this limits the ability to evaluate whether there is a compounding effect of consumption of multiple smokeless tobacco types in relation to risk of low birth weight of infants.

## Conclusion

The adverse effects of smokeless tobacco found in this review highlights the need for policies which focus on primary prevention of smokeless tobacco use. Pregnant women should be provided with intensive education, counselling and resources regarding quitting or cutting down smokeless tobacco use to improve both maternal and child health outcomes. However, this is a challenging matter due to the addictive nature of tobacco.

## Supporting information

S1 Checklist PRISMA 2020 checklist. (DOCX)

S1 File (DOCX)

S2 File (XLS)

S3 File (XLS)
