# Supplementary material for: The invisible enemy: A systematic review and meta-analysis of maternal smokeless tobacco use as a risk factor for low birth weight
Source: PLoS One. 2024 Dec 30;19(12):e0312297. doi: 10.1371/journal.pone.0312297 (PMC11684629; doi:10.1371/journal.pone.0312297)
Supplement: S1 File — (DOCX) [file pone.0312297.s002.docx]

**Ovid MEDLINE(R) <1946 to November 23, 2022>**

**1 pregnan*.mp. or exp Pregnancy/ 1068329**

**2 (prenatal or "pre natal" or mother or maternal or gestation or reproduc*).mp. [mp=title, book title, abstract, original title, name of substance word, subject heading word, floating sub-heading word, keyword heading word, organism supplementary concept word, protocol supplementary concept word, rare disease supplementary concept word, unique identifier, synonyms] 1454171**

**3 1 or 2 2064233**

**4 exp Tobacco, Smokeless/ 4039**

**5 (smokeless or chewing or chew or quid or dip or leaf or snus or snuf* or gutka or guthka or gudhaku or iqmik or gul or "pan masala" or mishri or misri or khaini or mawa or kima or shamma or tombak).mp. [mp=title, book title, abstract, original title, name of substance word, subject heading word, floating sub-heading word, keyword heading word, organism supplementary concept word, protocol supplementary concept word, rare disease supplementary concept word, unique identifier, synonyms] 103564**

**6 4 or 5 103564**

**7 exp Birth Weight/ 45274**

**8 exp Infant, Low Birth Weight/ 38262**

**9 7 or 8 76508**

**10 3 and 6 and 9 62**

Search strategy

<https://access.ovid.com/custom/redirector/index.html?dest=https://go.openathens.net/redirector/www.monash.edu?url=http://ovidsp.ovid.com/ovidweb.cgi?T=JS&NEWS=N&PAGE=main&SHAREDSEARCHID=5fdsvijmdEBzb6QFIHUE1x7zYcj5AyJ61UU5zf3st8XENYRlVGSyfylFsfpFQpLou>
